# Characterizing the plasma protein binding profiles of chemistry diversified antisense oligonucleotides in human and mouse plasma using an ultrafiltration method

**DOI:** 10.3389/fphar.2024.1481937

**Published:** 2025-01-22

**Authors:** Cassandra Yun, Kazuki Fukami, Raku Shinkyo, Rongrong (Rosa) Jiang

**Affiliations:** ^1^ Eisai, Cambridge, MA, United States; ^2^ Eisai Co., Ltd., Tsukuba, Japan

**Keywords:** antisense oligonucleotides, plasma protein binding, ultrafiltration, MOE, PMO, binding saturation, γ-globulins

## Abstract

**Introduction:**

Plasma protein binding plays a significant role in influencing the pharmacokinetic and pharmacodynamic properties of drugs. This study focuses on examining two pairs of sequence-matched ASOs: phosphorodiamidate morpholino oligomers (PMOs) and 2’-O-methoxyethyl/phosphorothioate (MOE/PS)-modified ASOs, to assess their plasma protein binding profiles.

**Methods:**

The binding of both PMO and MOE/PS-modified ASOs was investigated using an ultrafiltration method combined with hybridization electrochemiluminescence, allowing for the measurement of the unbound fraction (*f*u) in both mouse and human plasma. To further characterize the interaction between ASOs and plasma proteins, individual binding measurements were taken for five major proteins in human plasma: human serum albumin, α1-acid glycoprotein, human γ-globulin, low-density lipoprotein, and high-density lipoprotein.

**Results:**

The results showed a notable difference in plasma protein binding between the two types of ASOs, with MOE/PS-modified ASOs exhibiting significantly higher binding compared to PMOs. The *f*u, plasma values revealed no significant species difference between mouse and human plasma. Additionally, a saturation point for *f*u, plasma was observed in MOE/PS-modified ASOs at concentrations above 1 μM, whereas PMOs did not show saturation even at concentrations up to 10 μM. Notably, human γ-globulins were found to have a predominant binding affinity for both MOE/PS and PMO ASOs at physiological concentrations, surpassing human serum albumin, the most abundant plasma protein.

**Discussion:**

The results suggest that the chemistries of the ASOs, particularly their modifications, are key determinants of their binding profiles. The study also highlights the important, though previously overlooked, role of human γ-globulins in the plasma protein binding of ASOs. This could have implications for understanding ASO distribution and tissue disposition, which may inform the development and optimization of ASO-based therapies.

## 1 Introduction

Phosphorothioate (PS) modified antisense oligonucleotides (ASO) are a widely used modality in therapeutic drug development for a broad range of diseases. In addition to PS backbones, 2′-ribose sugar modifications and sugar phosphate modifications improve nuclease-resistance, prolonged tissue retention, and binding affinity to the RNA target. These modifications can also influence the pharmacokinetics properties of ASOs. PS-ASOs have negatively charged backbones known to have high protein binding capacity and improved plasma stability (Brown et al., 1994). Further 2′-O-methoxyethyl (MOE) modification of the sugar (ribose) can increase protein binding affinity of PS-ASOs (Geary et al., 2001). Neutrally charged antisense agents such as phosphorodiamidate morpholino oligomer (PMO) replace the pentose sugar with a morpholine ring, and the phosphate with a neutral phosphorodiamidate linkage. PMOs bind less strongly to plasma proteins and are more readily filtered and excreted from the body, resulting in lower tissue uptake and low protein interactions (Amantana and Iversen, 2005).

Plasma protein binding (PPB) properties of antisense oligonucleotides are crucial to understand pharmacokinetics, which can have important implications involving drug distribution and potential drug toxicity. A detailed understanding of plasma protein binding profile of ASO therapeutics can therefore facilitate the construction of pharmacokinetic and pharmacodynamic (PK/PD) relationship and further predict therapeutic index. Many techniques and methodologies have been developed and used to measure the unbound fraction (*f*u) for small molecules. Among these are equilibrium dialysis, ultrafiltration (Pacifici and Viani, 1992). ultracentrifugation (Brockman et al., 2015), equilibrium gel filtration (Weiss and Gatlik, 2014). Equilibrium dialysis is employed most frequently for small molecule PPB investigation (Di et al., 2017), followed by ultrafiltration. However, due to the uniqueness of physicochemical properties of ASOs, such as relatively high molecular weight, linear structure, and nonspecific binding, ASOs present unique challenges in ƒu determination with traditional techniques. Because of the potential linear conformation of ASOs, the molecular weight cutoff (MWCO) for dialytic membranes needs to be much higher than the ASO molecular weight (generally up to 7 KDa). The lack of commercially available equilibrium dialysis membranes with MWCO over 20 KDa makes this technique incompatible with ASOs (Rocca et al., 2019). Ultracentrifugation employs the use of high speed and although advantageous in the reduction of non-specific binding to the surface due to the lack of membrane, the method can be costly and low throughput compared to ultrafiltration (Guimaraes et al., 2022). This makes ultrafiltration the more feasible methodology for *f*u measurement for ASOs. Ultrafiltration method also has a membrane portion which becomes a major source for nonspecific adsorption and low recoveries of ASOs. To overcome this issue, some reports that membrane pretreatment with surfactants or sacrificial oligonucleotides can successfully mitigate nonspecific binding and improve recovery. Due to chemical diversity and binding differences of ASO analogs, one needs to modify pretreatment conditions and measure recovery of individual ASO in the testing system to ensure the non-specific binding is mitigated under assay condition.

In this paper, ƒu of ASO in plasma was evaluated using an ultrafiltration method. The assay condition of ultrafiltration method was optimized to each ASO. To assess the effect of chemistry and length of ASO, *f*u of two pairs of sequence matched MOE/PS and PMO-modified ASO were evaluated in mouse and human plasma at multiple concentrations. To further explore the impact of PPB in ASOs, we identified the major binding proteins for MOE/PS and PMO ASOs in human plasma. The results and its potential impact are further explored in this paper.

## 2 Materials

ASOs were purchased from Gene Tools (Philomath, OR) and Wuxi AppTec (China). Sequence-specific detection and capture probes conjugated with biotin and digoxigenin, respectively, were purchased from Integrated DNA Technologies (Coralville, IA). The two pairs (25mer-PMO/25mer-MOE and 20mer-PMO/20mer-MOE) each have identical sequences and length, but different chemical modifications.

(*S*)-Warfarin and Antipyrine were purchased from FUJIFILM Wako Pure Chemical Corporation (Osaka, Japan) and Sigma-Aldrich (St Louis, MO), respectively.

Nanosep 0.5-mL centrifugal filters [30K MWCO] were purchased from Pall Corporation (Westborough, MA), Tween-20, and Tween-80 were purchased from Millipore Sigma (St Louis, MO). Polyethylene tubes were obtained from ASIAKIZAI Inc. (Tokyo, Japan) and siliconized tips were obtained from Thomas Scientific (Swedesboro, NJ).

Human serum albumin (HSA), α1-Acid glycoprotein from human plasma (α1-AGP), human γ-Globulins (HG), Lipoprotein-low density from human plasma (LDL), and Lipoprotein-high density from human plasma (HDL) were all purchased from Millipore Sigma.

Mouse plasma samples (Na Heparin treated) were from frozen pooled, mixed-gender donors from CD-1 mice and human plasma samples (Na Heparin treated) were from frozen pooled, mixed-gender donors obtained from BioIVT (Westbury, NY). For small molecule method qualification, human plasma (Na Heparin treated) was collected from male volunteers. Blocker Casein in TRIS-buffered saline (TBS), Tris-EDTA (TE) Buffer, and phosphate-buffered saline (PBS) were obtained from Thermo Fisher Scientific (Waltham, MA). MSD Read Buffer T (4X) with Surfactant was obtained from Meso Scale Diagnostics (Rockville, MD).

## 3 Methods

### 3.1 Sample preparation

ASO stock solutions were prepared in PBS, 0.1% Tween-20 and 1M NaCl in TE Buffer (0.1% Tween-20), mouse plasma, or human plasma.

#### 3.1.1 Evaluation of non-specific binding

Non-specific binding (NSB) is the binding of the target ASO to non-targeted sites when contact is made with a surface, and it is important to control this parameter to ensure maximum recovery of the target analyte. Tween-20 is a non-ionic surfactant that can inhibit the binding of non-targeted sites to the target analyte and was therefore used as a control matrix to assess recovery (Batteiger et al., 1982). During method development, a 70% recovery cut-off was set at all concentrations tested and any result below the cut-off was indicated as a non-specific loss of analyte in the selected consumable. Recovery was calculated by comparing the pre-incubation sample in PBS to the sample in 0.1% Tween-20. All combined devices and consumables used to generate data reported in this study achieved a recovery higher than 70%.

#### 3.1.2 Matrix effect

The matrix effect was evaluated by taking blank matrix samples and applying ultrafiltration to obtain the pre-sample and post-bottom sample solutions. Each solution was spiked with 25mer-PMO (Supplementary Figure S1) and 20mer-MOE (Supplementary Figure S2) to create calibration curves. There was no signal difference between the pre-sample and post-bottom matrix.

### 3.2 Ultrafiltration

#### 3.2.1 Validation of the method

To validate the ultrafiltration method, small molecule compounds S-Warfarin and Antipyrine were used before testing the target ASOs. These compounds serve as standard reference molecules (Guimaraes et al., 2022), ensuring that the method is reliable and reproducible when applied to other molecules. The assay conditions for S-Warfarin and Antipyrine are the same as the testing ASO compounds described.

#### 3.2.2 Pre-treatment of filters

Before the sample is loaded onto the filters, it is critical to ensure that the filters are free of contaminants and non-specific binding (NSB) from previous samples or reagents. To achieve this, filters are pretreated by rinsing them with solutions that reduce such interference. The filters were pre-washed using either 300 µL of Milli-Q water (pure water) or 0.5% Tween-80 (a nonionic detergent). Tween-80 is commonly used to block non-specific binding sites on the filter surface and to minimize the retention of analytes. This step is critical for ensuring that no unwanted interactions occur between the sample and the filter, which could skew the results.

The filters were incubated at room temperature for 15 min to allow the solutions to interact with the filter surface. Following this incubation, centrifugation (5,000 × g for 10 min at 20°C) was applied to remove the excess pre-treatment solution. This ensures that any residual Tween-80 or water is removed before the actual sample loading step, further reducing any potential interference. After the centrifugation, the remaining filtrate was discarded, and the filters were washed twice with 500 µL of water to ensure no residual pre-treatment solution remains. After the wash, 300 µL of PBS (phosphate-buffered saline) or a 10 µM non-targeting control (NTC) solution was added to the filter. The NTC solution contains a scrambled sequence of the target analyte and is used to assess recovery without introducing bias. After adding PBS or NTC, the sample was incubated and spun down again under the same conditions to prepare the filter for sample loading. The collection tube was then switched to a new polyethylene tube to minimize the possibility of non-specific binding during elution. The polyethylene tube material helps reduce interactions between the filtrate and the tube surface, contributing to more accurate elution of the sample.

#### 3.2.3 Sample preparation, incubation and sampling

Designated concentrations of the target analytes (ASOs: 0.1–10 µM or small molecules: 1 µM) are spiked into neat mouse or human plasma. The samples are then incubated in a CO_2_ incubator at 37°C for 30–60 min with shaking at 300 rpm. This incubation step ensures that the analytes are well-mixed with the plasma, allowing for accurate filtration and separation. The temperature and shaking conditions mimic physiological conditions, ensuring the relevance of the data to *in vivo* situations.

After incubation, a 300 µL aliquot of the prepared sample (ASO or small molecule solution) is transferred to the pre-treated filter. Prior to centrifugation, a 25 µL aliquot of the sample (pre-sample) is taken and mixed with 25 µL of 0.1% Tween-20. After centrifugation (1,500 × g for 5 min at ambient temperature), the remaining solution below the filter is sampled as the post-bottom sample. This post-sample represents the fraction of analyte that passed through the filter. The remaining sample volumes were also measured to ensure that the bottom filtrate did not exceed 20% of the total volume to avoid overestimating the *f*u plasma measurements (Zhang and Musson, 2006).

#### 3.2.4 Sample processing and analysis

Both the pre-sample and post-sample are analyzed to determine recovery rates and to calculate *f*u plasma. ASO samples and small molecule samples are analyzed by using hybridization electrochemiluminescence (hECL) method and liquid chromatography with tandem mass spectrometry (LC-MS/MS), respectively. For small molecule samples, a matrix-matching approach is employed to avoid matrix effect on accuracy of analysis. To do so, pre-samples are diluted with blank filtrate and post-samples are diluted with neat blank plasma.

To evaluate for initial recovery for ASO samples, the signal of the pre-sample in plasma was compared to the ASO standard solution (ASO-control) in 0.1% Tween-20:

$$\%\;recovery=\frac{\left[pre\;sample\right]}{ASO\;control}\times 100$$ (1)

Quantitation for the unbound fraction in plasma sample was performed by comparing the signal of the pre-sample and the signal of the post-bottom sample after centrifugation:

$$\%\;fu=\frac{\left[post\;bottom\right]\;}{\left[pre\;sample\right]}\times 100$$ (2)

### 3.3 Hybridization electrochemiluminescence

A calibration curve with an appropriate analytical range was run concurrently during initial testing in different sample matrices (post-bottom, pre-sample) to ensure linearity and to confirm the absence of matrix effects. The samples were diluted according to their initial concentration (i.e. 1 μM is 100-fold diluted, 0.01 µM is 10-fold diluted) with 0.1% Tween-20 prior to hybridization to confirm the signal fell within the linear range. Using a 96-well PCR plate, the samples were added 1:1 to 0.1 µM of detection probe then hybridized under these conditions: 95°C for 10 min, 37°C for 60 min, and hold at 4°C until ready to analyze.

Subsequently, the MSD Gold 96-well Streptavidin SECTOR plate was first incubated with Blocker Casein in TBS at room temperature for 1 h, and then washed and incubated with 0.2 µM of the capture probe at 37°C with a shaking speed of 300 rpm. Hybridized samples were added, and 1 μg/mL of ruthenium-labeled anti-digoxigenin antibody in 0.05% Tween-20 in TBS followed with a wash and a 60 min incubation prior to each step. After a final wash, MSD Read Buffer was added, and the plate was read on a Meso QuickPlex SQ 120 MM instrument.

### 3.4 LC-MS analysis for warfarin and antipyrine

Two small molecules, warfarin and antipyrine, with known high and low protein binding in mouse and human plasma were processed using our ultrafiltration method. The collected samples for small molecules were deproteinized with 200 μL of 70% acetonitrile/30% methanol containing niflumic acid as the internal standard. The mixture was centrifuged, and the resulting supernatant was filtered (Multi Screen Solvinert, Filter Plates, 0.45 µm Low-Binding Hydrophilic PTFE, Millipore) and analyzed using LC-MS/MS. The LC-MS/MS system consisted of Prominence system (Shimadzu Corporation, Kyoto, Japan) equipped with an L-column (5 μm, 2.1 mm × 150 mm, Chemicals Evaluation and Research Institute) and API-4000 (AB Sciex LLC, MA). The mobile phases for chromatography were (A) distilled water containing 0.02% formic acid and (B) acetonitrile containing 0.02% formic acid. The injection volume was 5 μL and the run time was 6.0 min using a flow rate of 0.5 mL/min. The temperature in the column heater and auto-sampler was set at 40°C and 4°C, respectively. The mass spectrometer was operated in positive electrospray ionization mode. The transition ions (mass-to-charge ratio; *m*/*z*) were 189.1 > 56.5 for antipyrine, 309.228 > 163.096 for warfarin, and 283.000 > 245.000 for niflumic acid. The peak area ratios of antipyrine and warfarin to the internal standard were calculated. Details of HPLC gradient conditions and MS conditions are summarized in Supplementary Tables S1, S2.

### 3.5 Preparation of protein solutions

Powder from HSA, α1-AGP, and HG stocks were measured and dissolved with PBS at 40, 1.0, and 15 mg/mL, respectively. Solution from LDL and HDL stocks were diluted with PBS to obtain 3.5 and 4.0 mg/mL, respectively (Narita, 1979). The solutions were prepared and stored at 4°C the day prior to analysis. Each protein solution was spiked with a final concentration of 0.1 µM of the target ASO and incubated at 37°C for 60 min with shaking at 300 rpm using a VWR microplate shaker prior to ultrafiltration.

The following equation was used to calculate the reconstructed *f*u,plasma in the individually assessed proteins:

$$fu\left(\%\right)=\frac{1}{\Sigma \left(\frac{f_{bi}}{f_{ui}}\right)+1}\times 100$$ (3)

The following equation was used to calculate the individual contributions of each protein:

$$Contribution\;ratio\;of\;the\;protein\;i\;\left(\%\right)=\frac{\left(\frac{f_{bi}}{f_{ui}}\right)}{\left(\frac{f_{b}}{f_{u}}\right)}\times 100$$ (4)

where f_bi_ and f_ui_ are the bound and unbound fraction of the drug to protein I, respectively. The f_b_ and f_u_ are the bound and unbound fraction of the drug in plasma, respectively (Narita, 1979).

## 4 Results

### 4.1 Method qualification

Previously published methods using ultrafiltration by Guilherme and coworkers (Guimaraes et al., 2022) reported an unbound fraction of 5.5 and ∼85% in mouse plasma and 1.1% and 92.8% in human plasma for warfarin and antipyrine, respectively. They also reported values of 4.3% in mouse plasma and 1.5% in human plasma for warfarin using equilibrium dialysis which is one of the gold standard methodologies to measure plasma protein binding in industry. Both *f*u values evaluated with our ultrafiltration method were comparable to previously reported values: 2.9% ± 0.49% and 80% ± 0.41% in mouse plasma, and 0.30% ± 0.0052% and 78% ± 2.1% in human plasma for warfarin and antipyrine, respectively (mean of triplicate and standard error of the mean).

### 4.2 Recovery assessments

Recovery assessments were done with our compounds in PBS to ensure minimal influence of NSB to the consumables used during analysis. Low-binding consumables were evaluated to confirm sufficient recovery of the target-ASO, and two filter pretreatment conditions were analyzed at concentrations ranging from 0.002 to 1 µM. If the recovery percentage (calculated based on Equation 1) fell below 70%, then the evaluating parameters used at that concentration was considered inadequate for future analysis if the unbound concentration of the target ASO also fell within the expected range. Given the sticky nature of ASOs, insufficient treatment of the filtration membrane prior to analysis will result in poor recovery and therefore inaccurate measurements of the unbound fraction of our compound.

Multiple pretreatment methods of the filtration membrane were tested at 1 µM: MilliQ water with PBS, 0.5% Tween-80 with PBS, and 0.5% Tween-80 with NTC (with the appropriate chemical modification). At a minimum, 0.5% Tween-80 was necessary to inhibit non-target binding sites, however, in some cases it was still inadequate. 20mer-MOE required additional filter treatment with NTC because 0.5% Tween-80 and PBS was not enough to mitigate NSB.

Once the filtration membrane pretreatment was established for each compound, multiple concentrations were evaluated to cover the *f*u range specific to the chemical modification (i.e., lower concentrations were evaluated for MOE/PS due to expected low unbound plasma concentration). As summarized in Table 1, 25mer-PMO and 20mer-PMO had good recovery at concentrations of 0.05 and 0.3 µM (or *f*u of 5.0% and 30%), respectively, which covers the projected unbound fraction of 62.7%–93.9% reported in clinically approved ASOs in human plasma as shown by Jiang and coworkers (Jiang et al., 2023). Likewise, the projected unbound fraction for MOE/PS was 3.9%–5.9% and the recovery concentrations of 0.002 and 0.01 µM (or *f*u of 0.50% and 1.0%) proved sufficient for 25mer-MOE and 20mer-MOE, respectively. The conditions used in this study suggest no recovery concern.

**TABLE 1:** Recovery (%) of four ASOs using different filter pretreatment conditions.

Recovery (%)					
Filter Pretreatment	Conc. (µM)	20mer-MOE	20mer-PMO	25mer-MOE	25mer-PMO
Water + PBS	1	66	-	-	17
0.5% Tween 80 + PBS	1	89	89	80	82
	0.3	-	89	-	-
	0.1	71	87	71	76
	0.05	63	-	83	76
	0.01	29	-	77	-
	0.005	-	-	76	-
	0.002	-	-	70	-
0.5% Tween 80 + NTC	1	83	-	-	-
	0.1	85	-	-	-
	0.05	85	-	-	-
	0.01	74	-	-	-

Recovery evaluation was done with target-ASO spiked in PBS, using nanosep (30K) filters, siliconized tips, and polyethylene (ASIAKIZAI) tubes (n = 3). Expected unbound concentrations in human plasma for 20mer-MOE, 20mer-PMO, 25mer-MOE, and 25mer-PMO, are greater than 0.01, 0.1, 0.002, and 0.05 µM, respectively. The recovery assessments for each individual target ASO, reflect good recovery (>70%) at their respective filter pretreatment requirements. (-) indicates that assay condition is not tested.

### 4.3 Unbound fraction measurement

Four compounds were evaluated for plasma protein binding using their respective ultrafiltration pre-treatment condition in mouse and human plasma. The two pairs (25mer-PMO/25mer-MOE and 20mer-PMO/20mer-MOE) each have identical sequences and length, but different chemical modifications. As summarized in Table 2, the *f*u (calculated by Equation 2) between the two matrices do not indicate a significant species difference respective to the ASO. However, we observed that the chemistry modification of MOE/PS also significantly influences the *f*u and higher binding is observed. The resulting *f*u for 20mer-MOE, 25mer-MOE, 20mer-PMO, and 25mer-PMO were 6.0, 1.3, 69, and 50% in mouse plasma and 4.4, 1.0, 56, and 36% in human plasma, respectively.

**TABLE 2:** Fu measurements of the four ASOs in mouse and human plasma.

ASO	Pre-treatment condition	*f*u (%), mouse plasma	*f*u (%), human plasma
20mer-MOE	Tween + NTC	6.0 ± 0.62	4.4 ± 0.14
20mer-PMO	Tween + PBS	69 ± 4.6	56 ± 5.5
25mer-MOE	Tween + PBS	1.3 ± 0.27	1.0 ± 0.17
25mer-PMO	Tween + PBS	50 ± 2.3	36 ± 4.3

All compounds listed above were evaluated using 1 µM of the target compound in their respective matrix (n = 3).

### 4.4 Binding saturation assessment

**FIGURE 1:**
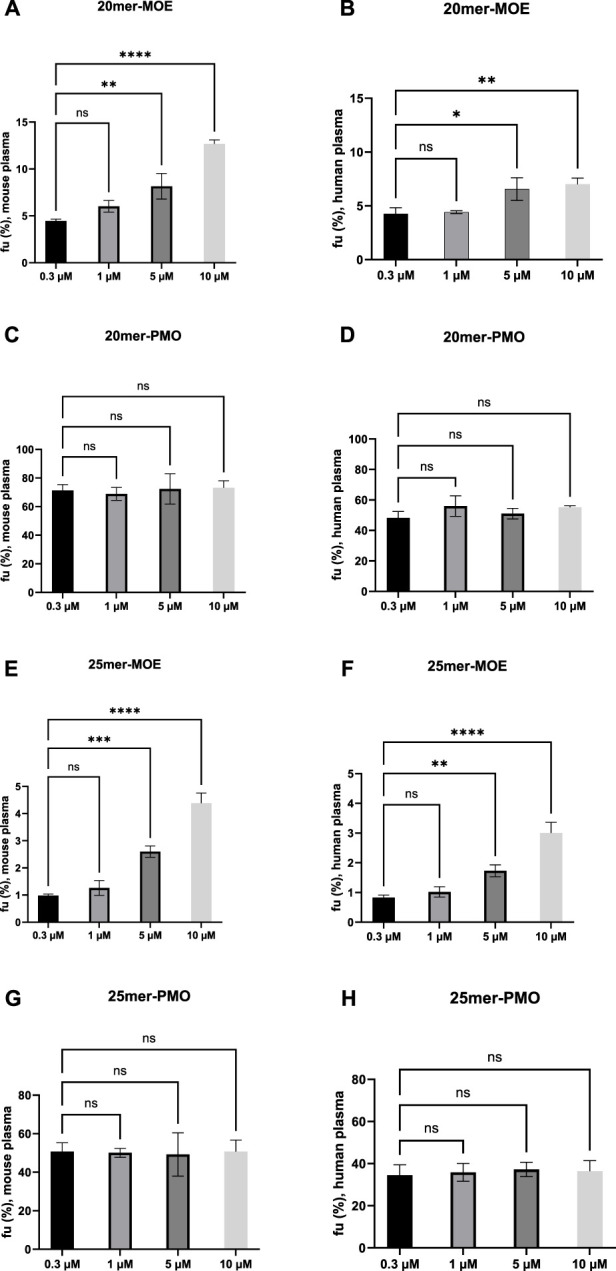
Trends of saturation of *f*u, plasma in MOE/PS and PMO in mouse and human plasma. Ordinary one-way ANOVA was used to analyze the differences among different concentration groups vs. 0.3 µM. Each bar represents the mean ± standard deviation of n = 3 samples. **(A, C, E, G)** are graphs showing results in mouse plasma. **(B, D, F, H)** are graphs showing results in human plasma. GraphPad Prism 10.0 software was used for statistical analysis and graphical representation. **(A, B, E, F)** No significant difference was observed in the mean values between 0.3 and 1 µM. Adjusted *P* values below 0.05 was considered to represent statistical significance: *P < 0.05, **P < 0.01, ***P < 0.001, ****P < 0.0001. **(C, D, G, H)** No significant difference was observed in the mean values between 0.3 µM and 1, 5, and 10 µM.

Limited capacity of the plasma protein to bind to the target drug can lead to saturation (Bohnert and Gan, 2013). A one-way analysis of variance (ANOVA) was applied across four different concentrations (0.3, 1, 5, and 10 µM) to determine if the binding of the target ASO saturates as concentrations are increased in mouse and human plasma (Figure 1). The analysis of these four concentrations was statistically notable across the different concentrations in varying degrees for 20mer- and 25mer-MOE. Saturation of binding is observed in both MOE/PS-modified ASOs at the given concentration range, whereas the binding of PMO-modified ASOs do not appear to saturate upon increasing concentrations up to 10 µM in either matrix. By contrast, the *f*u values of the MOE/PS-modified ASOs were increased dose-dependently where a positive correlation between *f*u and concentration is observed. Similar trends of saturation occur in both matrices and is consistent across the two species. There is no statistical significance when concentrations are increased from 0.3 to 1 µM and this observation occurs in both MOE/PS-modified compounds and in both matrices. Notable changes in *f*u are observed when concentrations increase from 0.3 µM to 5 and 10 µM. Based on our current data, this indicates that between 0.3 and 1 µM the protein binding is consistent, and when concentrations are increased beyond 1 μM, saturation of binding may occur for MOE/PS ASOs.

### 4.5 Major binding proteins in human plasma

Previous studies have demonstrated, by calculating the dissociation constant *K*_*d*_ or *K*_*m*_, that ASOs have a high capacity for binding to proteins, specifically albumin. (Srinivasan et al., 1995; Crooke et al., 2020). The high abundance plasma proteins known to bind to various molecules include HSA, α1-AGP, and HG, and we therefore diluted these three proteins into PBS at their physiological concentrations at 40, 1.0, and 15 mg/mL, respectively, to characterize the interaction for PMO and MOE/PS in human plasma. The plasma protein binding of 25mer-PMO and 25mer-MOE were reconstructed from the protein binding in each solution (Table 3) using Equation 3. The comparison of the measured *f*u,plasma of 25mer-PMO to the calculated value was considered to be reasonably reproducible using all three individual proteins (Table 3). This result indicates that HSA, α1-AGP, and HG are the major plasma proteins that contribute to 25mer-PMO binding in human plasma. The contribution ratios in each protein were calculated based on Equation 4 as 22% for HSA, 20% for α1-AGP, and 58% for HG, respectively (Table 4), therefore indicating HG as the primary contributor to the binding of 25mer-PMO. For 25mer-MOE, the calculated value from all three proteins was 2.0%, which is two-fold higher than the measured *f*u,plasma of 1.0%. This suggests that the major protein binding profile may be incomplete. Two additional protein solutions, LDL and HDL, were later included at 3.5 and 4.0 mg/mL, respectively, to complete the protein binding assessment for *f*u,plasma of 25mer-MOE. These 5 proteins were estimated at *f*u,plasma of 1.3%, which is closer aligned to the measured value of 1.0% (Table 3). The contribution ratios in each protein were 13% for HSA, 2.0% for α1-AGP, 50% for HG, 18% in LDL, and 17% in HDL, respectively (Table 4). Like 25mer-PMO, HG is the primary protein that contributes to most of the binding of 25mer-MOE.

**TABLE 3:** Measured unbound fraction of 25-mer-PMO and 25mer-MOE in five different human plasma proteins.

Individually Assessed Proteins	Measured *f*u, protein (%)	
	25mer-PMO	25mer-MOE
Human Serum Albumin (HSA), 40 mg/mL	62	9.0
α1-Acid Glycoprotein (α1-AGP), 1.0 mg/mL	64	37
γ-Globulins (HG), 15 mg/mL	38	2.6
Lipoprotein, low density (LDL), 3.5 mg/mL	-	7.0
Lipoprotein, high density (HDL), 4 mg/mL	-	7.3
Calculated *f*u,plasma from individually assessed proteins (HSA, α1-AGP, HG)	26	2.0
Calculated *f*u,plasma from individually assessed proteins (HSA, α1-AGP, HG, LDL, HDL)	-	1.3
Measured *f*u,plasma at 1 µM	36	1.0

All protein solutions (5) listed above were evaluated using 0.1 µM of the target compound in their respective solution(s) (n = 3). *f*u,plasma values were calculated using Equation 3 as described in Methods.

**TABLE 4:** Calculated contribution for 25mer-PMO and 25mer-MOE from five different human plasma proteins.

Individually assessed proteins	Contribution	
	25mer-PMO	25mer-MOE
Human Serum Albumin (HSA), 40 mg/mL	22%	13%
α1-Acid Glycoprotein (α1-AGP), 1.0 mg/mL	20%	2.0%
Human γ-Globulins (HG), 15 mg/mL	58%	50%
Lipoprotein, low density (LDL), 3.5 mg/mL	-	18%
Lipoprotein, high density (HDL), 4 mg/mL	-	17%

Contribution values were calculated using Equation 4 as described in Methods.

## 5 Discussion

Historically, *f*u determination for small molecule drug candidates has been determined by ultrafiltration, ultracentrifugation, or equilibrium dialysis. Owing to ASOs’ physicochemical characteristics, such as a relatively high molecular weight, linear structure, and nonspecific binding, ASOs present unique challenges in *f*u determination through traditional techniques. In the current study, we employed an ultrafiltration-based approach and optimized it by selecting appropriate MWCO for the filtration membrane and filtration device with lower adsorption of ASO analytes by pre-treating the filtration membrane to minimize NSB. The validity and robustness of our assay system is confirmed with reported *f*u values of warfarin and antipyrine with mouse and human plasma. The *f*u values of MOE/PS and PMO generated using our method were similar with reported values compared with nusinersen (full MOE, 18mer, *f*u,human plasma of 3.9%–5.9%) and viltolarsen (full PMO, 21mer, *f*u,human plasma of 60%) (Shunji et al., 2023). Additionally, our results of the two sequence-matched MOE/PS and PMO pairs align very well with the understanding that the plasma protein binding of ASOs is primarily driven by their chemistries. This means that ASOs with the same sequence, but different chemistries can exhibit significantly different degrees of plasma protein binding, leading to varying levels of unbound drug concentration. Furthermore, our data suggested that species difference is considered as minor between human and mouse, with *f*u slightly higher in mouse plasma than that in human plasma for both MOE/PS and PMO ASOs. This finding aligns with the review paper (Crooke, 2007) with a conclusion that typically, in mouse, plasma protein binding is slightly less than that in non-human primates or humans.

Although MOE/PS ASOs have extensive PPB, to best of our knowledge, there is no clear evidence of competition between PS ASOs and other small molecule or ASO drugs (Crooke et al., 2020). For example, as reported by Watanabe and coworkers (Watanabe et al., 2006), there is no direct competition observed between phosphorothioate oligonucleotides ISIS 2302 and warfarin, which is a highly albumin bound SM. In addition, Watanabe et al. (2006) and Shemesh et al. (2016) concluded there is no concentration-dependent plasma protein saturation for the tested PS ASO or MOE gapmer ASOs, while another study conducted by Clement and coworkers (Clement et al., 2024) suggested a trend of *f*u elevation of a 20mer-MOE gapmer ASO when increasing from 0.8 to 25 µM with mouse and human plasma. Note the methodologies and concentration ranges evaluated in these studies are very different and thus may have impact on data interpretation. Here, we reported for the first time a concentration dependent saturation phenomenon for tested ASO, which is seen with MOE/PS ASO only but not PMO under the defined assay condition. As summarized in Figure 1 and Table 2, we confirmed that measured *f*u values of MOE/PS ASO at 0.3 and 1 µM were comparable and not statistically different, suggesting the *f*u values at 0.3 µM reflect plasma protein binding at non-saturable concentrations. Then, the saturation is judged by achieving statistical significance when compared between measured *f*u values at that concentration and the lowest measured concentration of 0.3 µM. At the concentration of 5 µM and above, we observed binding saturation of tested MOE/PS ASOs in both mouse and human plasma. Therefore, we consider our MOE/PS ASOs may start to demonstrate saturation when concentrations increase beyond 1 µM. This concentration-dependent saturation of MOE/PS ASO reported here may occur in the clinically relevant concentration ranges in human plasma. As summarized in Table 5, the reported clinical plasma concentrations of MOE/PS ASOs may reach to the micromolar range. For example, the reported C_max_ of Danvatirsen (a MOE/PS ASO) after 200 mg IV dosing is as high as 4.88 µM in human subjects. Additionally, there were a few reported C_max_ from clinical studies of other MOE/PS ASOs where they reach levels above 1 µM when dosed via varied administration routes. Furthermore, people should keep this saturation potential in mind from a preclinical development perspective as well, as PPB saturation might happen with *in vivo* pharmacological screen studies which are commonly conducted at supratherapeutic dose levels or with wide dose ranges. Such altered or dynamic PPB might have impacts on PK as well as PK/PD relationship by altering target tissue uptake or intracellular transport for MOE/PS ASOs.

**TABLE 5:** Reported human plasma C_max_ values for ASO therapeutics on the market and under clinical development.

Drug	Modification	Approved/Clinical trial	*f*u (%) in human plasma	Dose	C_max_ (µM)	References
Nusinersen	18-mer, MOE/PS	Approved	3.9–5.9	12 mg (IT)	0.116	Nusinersen (2017)
Mipomersen	20-mer, MOE-gapmer	Approved	4.13–15.19	200 mg (IV) 100–300 mg (SC)	2.5 0.5	Mipomersen (2012)
Inotersen	20-mer, MOE-gapmer	Approved	2.2–5.6	150 mg (SC) 300 mg (SC)	0.415 0.835	Inotersen (2017)
Danvatirsen	MOE/PS	Clinical Trial	N/A	3 mg/kg (IV) 200 mg (IV)	4.83 4.88	Xu et al. (2019)
Remlarsen^a^	MOE	Clinical Trial	N/A	5.3 mg (ID) 5.3 mg (ID, multiple)	0.0018 0.0047	
GSK3228836^b^	MOE-gapmer	Clinical Trial	N/A	150 mg (SC) 300 mg (SC)	0.83 2.04	

C_max_ values from approved drugs are cited from U.S. FDA (Non-Clinical and Medical Review(s)) review documents and PMDA, review reports. Cmax values from drugs currently in clinical trials are cited from reported results on clinicaltrials.gov. Intrathecal (IT); Intravenous (IV); Intradermal (ID); Subcutaneous (SC).

^a^ Remlarsen: Clinical Trial ID: NCT03601052, miRagen Therapeutics, https://clinicaltrials.gov/study/NCT03601052.

^b^ GSK3228836: Clinical Trial ID: NCT04449029, GlaxoSmithKline, https://clinicaltrials.gov/study/NCT04449029.

Understanding the interactions between ASOs and these major plasma proteins is crucial for studying the PK profile, optimizing the design and delivery of ASO-based therapeutics. Nevertheless, the extent of research in this area remains limited. Gaus and coworkers (Gaus et al., 2018) systemically evaluated the binding affinity of chemically modified PS-ASOs with several abundant human plasma proteins by size-exclusion chromatography. Among all protein tested, histidine-rich glycoprotein demonstrated the lowest K_d_ value of 0.009 µM, while the most abundant plasma protein albumin and second abundant protein IgG have reasonably low K_d_ value of 12.7 and 1.6 µM, respectively (Gaus et al., 2018). In the current study, we studied the binding interaction between test ASOs and major proteins existing in human plasma by measuring the *f*u value at their physiological protein concentrations. Our results clearly suggest that HSA, α1-AGP and HG proteins represent the major proteins responsible for binding to tested 25mer-PMO, while 25mer-MOE was found to be associated with all 5 proteins, namely, HSA, α1-AGP, HG, LDL, and HDL. Even though individual protein profiles might be different between MOE/PS and PMO ASOs, the protein that contributes most in terms of binding is the same for both MOE/PS and PMO. HG itself accounted for 58% and 50% binding contribution for tested PMO and MOE/PS ASOs, respectively (Table 4).

Given the high concentration of albumin in plasma and in common with most xenobiotics, people tend to consider ASOs bind extensively to plasma albumin and therefore albumin plays a critical role in PPB of ASOs (Crooke et al., 2020). However, our finding suggests for the first time that HG, rather than HSA might be the most critical driving force for plasma protein binding for ASO drugs in both PMO and MOE/PS chemistries. The highest contribution of HG in ASO plasma protein interaction reported here indicated that the field may have overlooked the role of HG in disposition of ASO drugs after administration and therefore posing the need for better understanding of such interaction and its potential impacts. For instance, it has been reported that enhancement of PPB may increase and facilitate target tissue distribution for ASOs, such as muscle targeting ASOs (Prakash et al., 2019). Albumin, LDL, and HDL as most abundant plasma proteins are being investigated and demonstrated “positive” impact to target tissue distributions. In a recent paper reported by Prakash et al. (2019), the authors hypothesized that improving albumin binding will facilitate traversal of ASO from the blood compartment to the interstitium of the muscle tissues to enhance ASO functional uptake. However, the potential of such albumin-facilitated cellular uptake is very much limited by the observation reported by Chappell and coworkers (Chappell et al., 2020) that albumin can also facilitate the transport of ASO from the interstitium to the lymph and back into circulation, which yield in only a slight overall increase in ASO activity (∼2-fold). The role of HG in plasma protein-facilitated cellular uptake is therefore yet to be investigated and may provide another mechanism insight to plasma protein association enhanced cellular uptake and a novel target for optimization of such interaction. Additionally, HG are immunoglobulins comprising five classes: IgM, IgG, IgA, IgE, and IgD, the concentrations or patterns of which can be altered by various conditions such as age (Buckley and Dorsey, 1971), gender (Cassidy et al., 1974), smoking condition (Tarbiah et al., 2019) and disease status, etc. Changes in HG levels may impact the overall plasma protein binding capacity and distribution of ASO therapeutics, potentially influencing their PK and PD. For instance, patients with chronic liver disease (Liu et al., 2015), certain types of cancer (Gerçel-Taylor et al., 2001; Liu et al., 2015), or hypergammaglobulinemia (Zhao et al., 2021) are reported to have altered HG levels. These patient populations therefore might exhibit a reduced or elevated *f*u and a different PPB profile for administrated ASO therapeutics compared to the healthy population. Understanding these differences will be important for decision making if tailoring dosing regimens and optimizing the efficacy of ASO therapeutics are warranted in patient populations.

Over the past 30 years, the field of oligonucleotide therapeutics has advanced sufficiently and ASOs are positioned as promising therapeutic modalities targeting a variety of diseases. While ASOs have demonstrated great biological potency against their targets, the target tissue distribution of ASOs is still considered insufficient and therefore remains as a key element for improving PD potential further. Interactions of chemically modified ASO therapeutics with plasma proteins play an important role in facilitating such distribution from the injection site to target tissues by reducing renal clearance and triggering PPB mediated cellular uptake. A robust assay system to accurately and quantitatively evaluate such ASO-plasma protein interaction and the knowledge generated hereby are important. These findings reported in our study might be applied to MOE/PS and PMO ASOs that have similar modifications and lengths compared to the ones reported in our study. Recently, there has been significant advancement in ASO delivery technology to improve target distribution. Such technologies include conjugating a naked ASO to peptides, lipids etc. The conjugated portion may or may not significantly change the lipophilicity and charge status of an ASO compound and results in alteration of their PPB profile. Similar studies are warranted with conjugated ASOs to better understand interactions and their significance in PK, PD, and toxicity of nucleic acid therapeutics with novel delivery technologies.

## 6 Conclusion

In this study, we systemically studied plasma protein binding profiles of ASOs employing two major chemistries (MOE/PS and PMO ASOs). Data suggested MOE/PS ASOs have saturation potential of plasma protein binding at clinically relevant exposure level. Additionally, our results suggested the important role of HG in interaction between plasma proteins and ASO therapeutics. The outcomes reported here can provide insights into the interaction of this novel modality with plasma proteins, enabling a better understanding of their pharmacokinetics characteristics, advancing the field of drug discovery, and development for ASO therapeutics.

## Data Availability

The original contributions presented in the study are included in the article/Supplementary Material, further inquiries can be directed to the corresponding authors.
