# Supplementary material for: Characterizing the plasma protein binding profiles of chemistry diversified antisense oligonucleotides in human and mouse plasma using an ultrafiltration method
Source: Front Pharmacol. 2025 Jan 22;15:1481937. doi: 10.3389/fphar.2024.1481937 (PMC11795045; doi:10.3389/fphar.2024.1481937)
Supplement: Supplementary file 1 [file DataSheet1.pdf]

Characterizing the Plasma Protein Binding Profiles of Chemistry Diversified Antisense Oligonucleotides in Human and Mouse Plasma Using an Ultrafiltration Method

Authors: Cassandra Yun[1], Kazuki Fukami[2], Raku Shinkyo[1]*, Rongrong Jiang[1]*

[1] Drug Metabolism and Pharmacokinetics, Eisai Inc., Massachusetts, USA

[2] Global Drug Metabolism and Pharmacokinetics, Eisai Co., Ltd. Tokodai 5-1-3, Tsukuba, Ibaraki 300-2635, Japan

*Correspondence

Rongrong Jiang, PhD, Drug Metabolism and Pharmacokinetics, Eisai Inc., Cambridge Massachusetts, USA

E-mail: Rongrong_jiang@eisai.com

Raku Shinkyo, PhD, Drug Metabolism and Pharmacokinetics, Eisai Inc., Cambridge Massachusetts, USA

E-mail: Raku_shinkyo@eisai.com

Key words: Antisense oligonucleotides, plasma protein binding, ultrafiltration, MOE, PMO, binding saturation, γ-Globulins

Supplementary Material

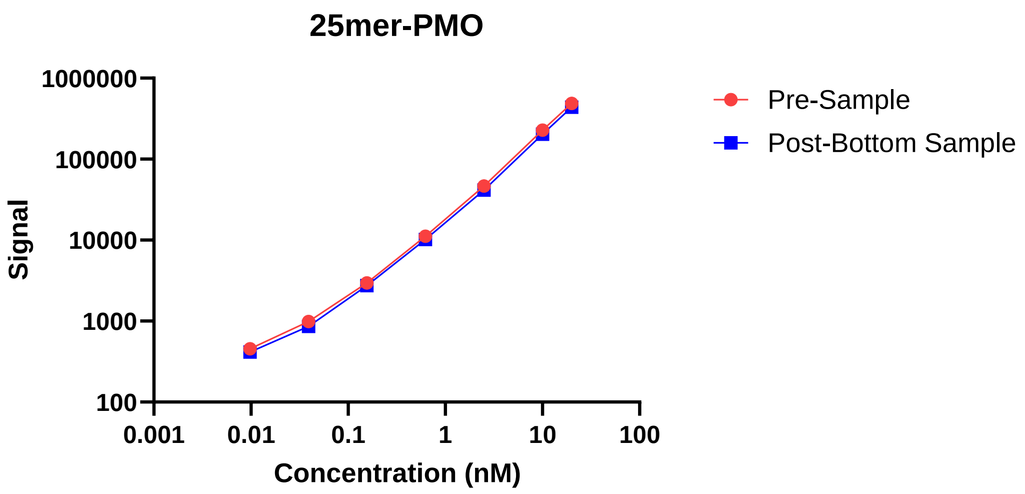

Supplemental Figure 1

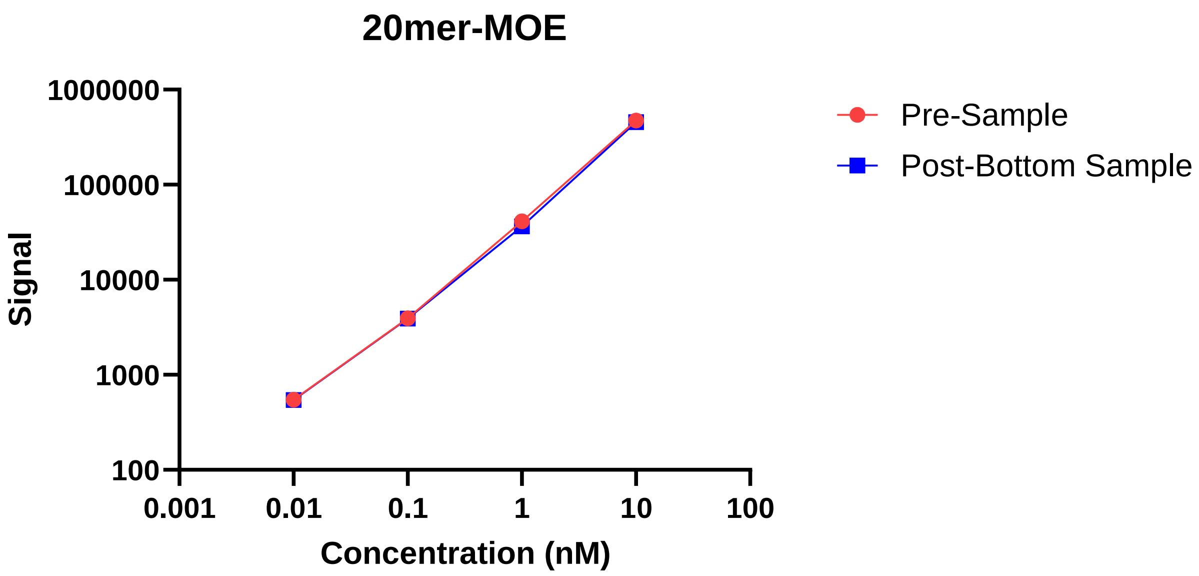

Supplementary Figure 2

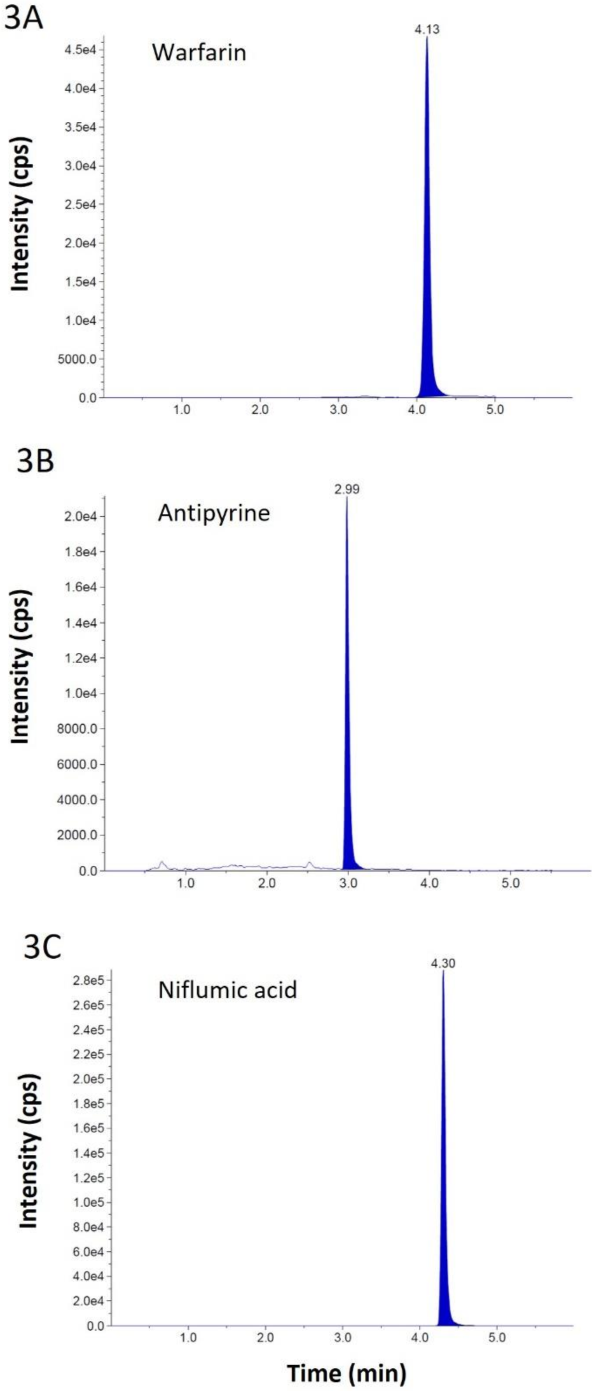

Supplementary Figure 3

Supplementary Figure 1 | 25mer-PMO Matrix Effect Blank pre-sample and post-bottom sample matrix spiked with 25mer-PMO at 0.00977, 0.0391, 0.156, 0.625, 2.50, 10.0, and 20 nM. There was no signal difference between the pre-sample or post-bottom sample solution in 25mer-PMO.

Supplementary Figure 2 | 20mer-MOE Matrix Effect Blank pre-sample and post-bottom sample matrix spiked with 20mer-MOE at 0.01, 0.1, 1, and 10 nM. There was no signal difference between the pre-sample or post-bottom sample solution in 20mer-MOE.

Supplementary Figure 3 | Representative chromatograms for small molecules (3A) Warfarin, (3B) Antipyrine and (3C) Niflumic acid.

Supplementary Table 1. HPLC gradient conditions for warfarin and antipyrine

**Time (min)**	**Mobile phase B**	**Flow rate (mL/min)**
	Warfarin	
0.5	2	0.5
2	99	0.5
4.5	99	0.5
4.51	2	0.5
6	2	0.5
	Antipyrine	
0.5	2	0.5
3	99	0.5
5	99	0.5
5.05	2	0.5
6	2	0.5

Supplementary Table 2. Multiple reaction monitoring (MRM) conditions for warfarin, antipyrine and niflumic acid.

**Precursor ion (*m/z*)**	**Product ion (*m/z*)**	**DP (V)**	**CE (V)**	**CXP (V)**
Warfarin				
189.100	56.500	40	50	11
Antipyrine				
309.228	163.096	50	20	12
Niflumic acid				
283.000	245.000	40	40	12

DP: Declustering potential

CE: Collision energy

CXP: Collision cell exit potential
